# A Survey on Deep Learning Based Segmentation, Detection and Classification for 3D Point Clouds

**DOI:** 10.3390/e25040635

**Published:** 2023-04-10

**Authors:** Prasoon Kumar Vinodkumar, Dogus Karabulut, Egils Avots, Cagri Ozcinar, Gholamreza Anbarjafari

**Affiliations:** 1 iCV Lab, Institute of Technology, University of Tartu, 50090 Tartu, Estonia; prasoon.vinodkumar@ut.ee (P.K.V.); dogus.karabulut@ut.ee (D.K.); egils.avots@ut.ee (E.A.); chagri.ozchinar@ut.ee (C.O.); 2 PwC Advisory, 00180 Helsinki, Finland; 3 iVCV OÜ, 51011 Tartu, Estonia; 4 Institute of Higher Education, Yildiz Technical University, Beşiktaş, Istanbul 34349, Turkey

**Keywords:** deep learning, 3D object recognition, 3D object segmentation, 3D object detection, 3D object classification

## Abstract

The computer vision, graphics, and machine learning research groups have given a significant amount of focus to 3D object recognition (segmentation, detection, and classification). Deep learning approaches have lately emerged as the preferred method for 3D segmentation problems as a result of their outstanding performance in 2D computer vision. As a result, many innovative approaches have been proposed and validated on multiple benchmark datasets. This study offers an in-depth assessment of the latest developments in deep learning-based 3D object recognition. We discuss the most well-known 3D object recognition models, along with evaluations of their distinctive qualities.

## 1. Introduction

3D object identification based on point clouds is a crucial component of a wide range of real-world applications, including autonomous navigation, housekeeping robots, reconstruction of architectural models of buildings, face recognition, preservation of endangered historical monuments, the creation of virtual worlds for the film and video game industries and augmented/virtual reality. In comparison to image-based detection, LiDAR (Light Detection and Ranging) delivers consistent depth information that may be utilised to correctly locate and classify objects. By utilising its active sensor, LIDAR can properly estimate range, which is becoming increasingly crucial in the perception system of current autonomous cars and robotics. LiDAR semantic segmentation seeks to estimate the labels for each point, which is essential for the perception system to comprehend its surroundings. Some of the LiDAR-based 3D recognition methods included in this survey are listed in Table 1. The accessibility of affordable sensors like the Microsoft Kinect has also made it possible for consumers to get short-range indoor 3D data and nowadays structure from motion (SfM) photogrammetry and neural radiance fields (Nerf) are becoming more popular. The direct acquisition of 3D data from the sensors is one of the main advantages of motion capture which makes it possible to get results relatively faster, sometimes even in real time. Thus, real-time motion capture of fast-moving objects is accomplished.

Identifying 3D objects from visual data has always been difficult. A scene may be recorded as 3D point clouds using 3D scanning tools like LiDAR or RGB-D sensors. Nevertheless, unlike pictures, LiDAR point clouds are sparse and have a highly varied point density due to factors such as non-uniform 3D sampling, the effective range of the sensors, occlusion, and relative position. It is difficult to conduct scene interpretation on LiDAR sequences due to the disorder and irregularity in the point cloud. The majority of algorithms currently in use only utilize the 2D information observed in RGB images to estimate the 3D bounding boxes by constructing pipelines from 2D data. These techniques result in a significant trade-off between efficiency and efficacy as they require numerous post-processing steps to combine predictions and delete unnecessary boxes. As an alternative to conventional 2D-based techniques, several methods employ 3D recognition techniques including segmentation, detection, and classification of 3D objects to add more 3D computations to the object detection pipeline.

A fundamental and complex task in computer vision and graphics is the segmentation and classification of 3D scenes. Building computer methods that identify the fine-grained labels of objects in a 3D environment is the goal of 3D segmentation, which has a variety of applications including autonomous driving, mobile robotics, industrial control, augmented reality, and medical picture analysis. 3D Object Segmentation can be further classified into three categories: Semantic segmentation to identify the labels for object classes like table and chair; Instance segmentation to make a distinction between various occurrences of the same class labels; and Part segmentation to further break down instances into their various parts, such as the armrests, legs, and backrest of a single chair. Due to the fact that 3D data, such as RGB-D, point clouds, projected pictures, voxels, and mesh, contain richer geometric, shape, and scale information with less background noise than 2D data, 3D segmentation provides a more thorough understanding of a scene than 2D segmentation. The majority of 3D systems employ two-stage methods to detect 3D objects, much like 2D image-based object systems: first, they create proposals, and then they perform detection. The 3D detection framework is simultaneously made more complex and more intriguing by the special characteristics of 3D systems, such as various data formats and the availability of both 2D and 3D images.

Approaches to 3D object classification continue to advance significantly in the deep learning era. Deep learning methods have recently taken the lead in numerous academic fields, including computer vision, speech recognition, and natural language processing. Deep learning for 3D object recognition has seen an increase in interest from the research community over the past ten years, driven by its success in learning potent features. However, there are still a lot of problems with 3D deep learning techniques. For instance, it can be challenging to combine characteristics from the RGB and depth channels. It is challenging to use local features in point clouds due to their irregularity, and transforming them into high-resolution voxels is quite computationally intensive. Despite 2D image detection, recognition, segmentation, and classification tasks being quite successful, using deep learning on 3D data is still difficult due to the sparse nature of most 3D data.

**Figure 1:**
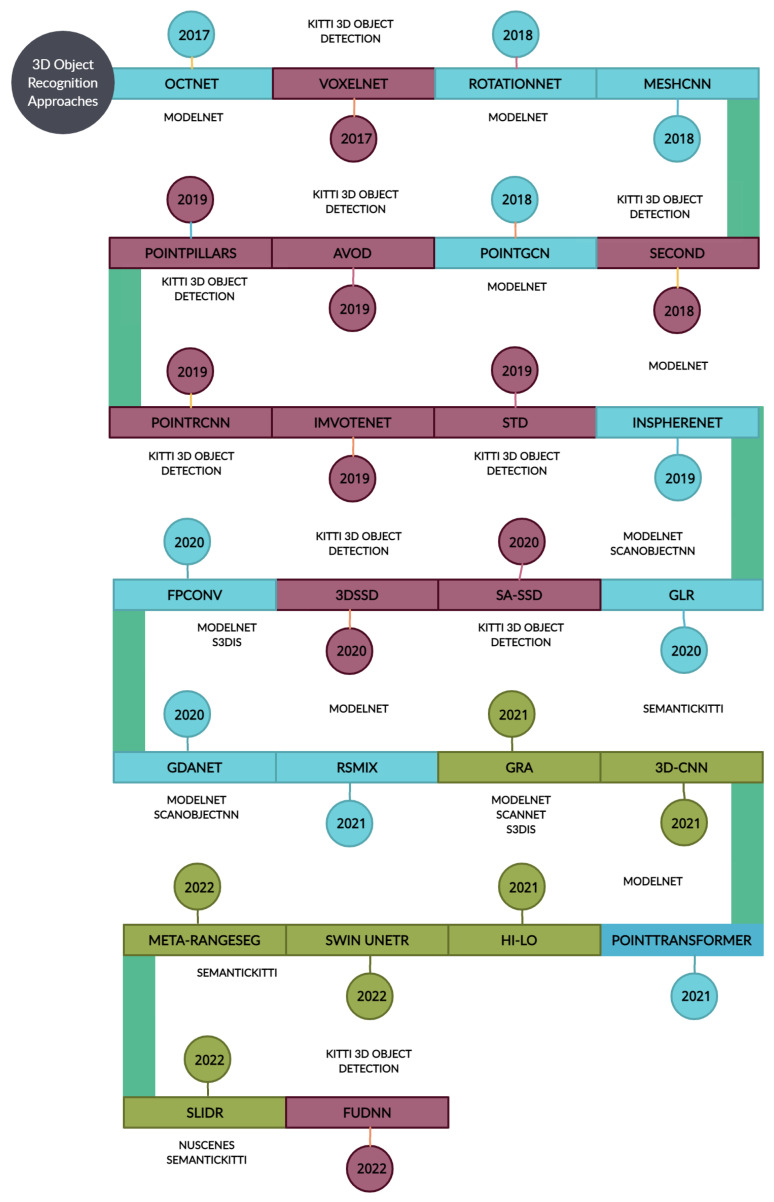
Timeline of different 3D Object Recognition Techniques discussed in this survey and the dataset they were evaluated based on their year of publication.

This study offers a thorough analysis of current developments in 3D object recognition using deep learning techniques including the benchmarking models, such as VoxelNet [1], OctNet [10], etc. It concentrates on examining frequently employed building components, convolution kernels, and full architectures, highlighting the benefits and drawbacks of each model. Over 33 representative papers that include 26 benchmark and state-of-the-art models and 7 benchmark datasets that have been used by many models over the last five years are included in this study. Despite the fact that certain notable 3D object recognition surveys, such as those on RGB-D semantic segmentation and point cloud segmentation, have been published, these studies do not exhaustively cover all 3D data types and common application domains. Most importantly, these surveys only provide a general overview of 3D object recognition techniques, including some of their advantages and limitations. Figure 1 shows the timeline of the different 3D Object Recognition approaches that were included in this survey, based on their year of publication. The figure also shows the dataset with which the performance of the models was evaluated.

The models surveyed in this article are selected depending on parameters like the dataset the models have been trained and/or evaluated upon, the method category they belong to, and the function they perform including, classification, segmentation, etc. Most of these models have used some benchmark datasets, like, SemanticKITTI [11] and Stanford 3D Large-Scale Indoor Spaces (S3DIS) [12] to validate and compare their performances with state-of-the-art technologies. Therefore, this study discusses some of the benchmark deep learning methods for 3D object recognition, and the main contributions are as follows:

- This work thoroughly discusses some of the state-of-the-art and/or benchmarking deep learning techniques for 3D object recognition, which includes segmentation, object detection, and classification, by utilizing a variety of 3D data formats, including RGB-D (IMVoteNet) [13], voxels (VoxelNet) [1], point clouds (PointRCNN) [3], mesh (MeshCNN) [14] and 3D video (Meta-RangeSeg) [15].
- We provide an extensive analysis of the relative advantages and disadvantages of different types of 3D object identification methods.
- Our work places special emphasis on deep learning techniques created expressly for 3D object recognition, including 3D segmentation, detection and classification.

## 2. Datasets

There are different benchmarking datasets that can be utilised to evaluate and improve the performance of deep learning models in 3D object recognition. These datasets contain scans of real-world objects which could include scenes from indoor and outdoor images. The datasets discussed in this survey are some of the benchmark datasets that are currently being used by many 3D object recognition methods. Only the datasets that have been used by the 3D object identification methods discussed in this survey paper in Section 3 (3D Segmentation), Section 4 (3D Detection), and Section 5 (3D Classification) will be listed. This includes the KITTI 3D Object Detection [16], SemanticKITTI [11], ModelNet10 and ModelNet40 [17], (S3DIS) [12], (nuScenes) [18], ScanNet [19] and ScanObjectNN [20] datasets. Datasets that are specific only to some 3D recognition methods, for example, 3D-CT dataset which is specific to HiLo-Network [21] or Multi-view images of rotated objects (MIRO) which is specific to RotationNet [22], will not be included in this survey. Table 2 provides the properties of data provided by different datasets.

### 2.1. KITTI 3D Object Detection

This benchmark dataset’s creators generated unique demanding datasets for stereo, optical flow, visual odometry/SLAM, and 3D object detection tasks. The 3D object dataset [16] focuses on object detection and 3D orientation estimation using computer vision techniques. This data is collected by manually categorising items in the Velodyne system’s 3D point clouds of the authors and projecting them back into the picture. This produces tracklets with precise 3D poses, which may be used to evaluate the effectiveness of 3D orientation estimation and tracking algorithms. Annotators were engaged to assign tracklets in the form of 3D bounding boxes to objects such as vehicles, vans, lorries, trams, pedestrians, and bicycles in order to build 3D object ground truth. This was accomplished by developing a special-purpose labelling tool that shows 3D laser pointers as well as camera pictures to improve annotation quality. The number of nonoccluded items in the picture, as well as the entropy of the object orientation distribution, are used to choose this dataset. High entropy is desirable for ensuring variety. This dataset contains 12,000 photos and 40,000 objects. The following methods discussed in this survey have validated their performances on this dataset: VoxelNet [1], SECOND [2], PointPillars [5], SA-SSD [6], STD [4], PointRCNN [3], 3DSSD [23], AVOD [9] and FuDNN [24]. Table 3 shows the comparison of the performance of these models on this dataset. The performance is evaluated in average precision (AP). The comparison is made based on the results published by the developers of these models. The table shows the average precision (AP) of models evaluated on the car class of the KITTI validation set. The results were evaluated with an IoU threshold of 0.7.

### 2.2. SemanticKITTI

SemanticKITTI [11] is a big dataset with remarkable detail in point-wise annotation and 28 classifications that may be used for a variety of purposes. This dataset’s authors concentrated on laser-based semantic segmentation and semantic scene completion. The collection differs from previous laser datasets as it contains exact scanwise annotations of sequences. Ultimately, all 22 sequences of the KITTI Vision Benchmark’s odometry [16], totaling over 43,000 scans, have been annotated. Furthermore, the revolving laser sensor’s whole horizontal 360-degree field of view has been labelled. This massive dataset was developed to inspire the creation of innovative algorithms, allowing researchers to study new research avenues and improve the assessment and comparison of these unique algorithms. This dataset is based on the KITTI Vision Benchmark’s odometry dataset [16], which depicts inner city traffic, residential neighbourhoods, motorway scenes, and country roads in and around Karlsruhe, Germany. The original odometry dataset comprises 22 sequences, with sequences 00 to 10 serving as the training set and sequences 11 to 21 serving as the test set. The same division has been used for this training and test set to maintain consistency with the original benchmark. Moreover, by giving labels exclusively for the training data, the original odometry benchmark is not altered. Altogether, this dataset has 23,201 complete 3D scans for training and 20,351 for testing, making it by far the biggest publicly available dataset. The following models discussed in this survey have validated their performances on this dataset: 3D-CNN [25], Meta-RangeSeg [15] and SLidR [7]. It was difficult to compare these models as the results published in the original paper were with different metrics.

### 2.3. ModelNet

ModelNet [17] is a large-scale object collection of 3D computer graphics CAD models, created by combining 3D CAD models obtained from 3D Warehouse, 261 CAD model websites indexed with the Yobi3D search engine, common item categories searched from the SUN database [26] that contain at least 20 object instances per category, and models from the Princeton Shape Benchmark [27]. Several previous CAD datasets were limited in terms of both the number of categories and the number of instances per category. The authors carefully verified each 3D model and deleted unnecessary items, including floor and thumbnail pictures, from each CAD model such that each mesh model has just one object from the identified category. This dataset comprises 151,128 3D CAD models from 660 different item categories. ModelNet10 and ModelNet40 are the common datasets that have been used in research works. The following methods discussed in this survey have validated their performances on this dataset: GRA [28], OctNet [10], RotationNet [22], PointGCN [8], InSphereNet [29], FPConv [30], GLR [31], RSMix [32], GDANet [33] and Point Transformer [34]. Table 4 shows the comparison of accuracy of PointGCN [8], GLR [31], RSMix [32] and RotationNet [22] evaluated on ModelNet10 dataset. Results of OctNet [10] could not be compared as the developers did not use any metric to measure the performance of that model. Table 5 shows the comparison of accuracy of PointGCN [8], InSphereNet [29], FPConv [30], GLR [31], RSMix [32], GDANet [33], GRA [28] and RotationNet [22] evaluated on ModelNet40 dataset.

### 2.4. S3DIS

Stanford 3D Large-Scale Indoor Spaces (S3DIS) [12] dataset contains five large-scale indoor rooms from three separate buildings, each of which covers around 1900, 450, 1700, 870, and 1100 square metres (total of 6020 square meters). These sections have a variety of architectural styles and appearances and largely consist of office areas, educational and exhibition spaces, conference rooms, personal offices, lavatories, open spaces, lobbies, stairways, and corridors. One of the sections has numerous floors, whereas the others only have one. With the Matterport scanner, the full point cloud is created automatically without any operator interaction. There are 12 semantic elements identified, which include structural components (ceiling, floor, wall, beam, column, window, and door) as well as regularly encountered goods and furnishings (table, chair, sofa, bookcase, and board). These classes are finer-grained and more difficult than typical semantic indoor segmentation datasets. S3DIS features 271 scenes divided into six zones. It includes 13 different types of semantic labels for scene segmentation. The following methods discussed in this survey have validated their performances on this dataset: GRA [28] and FPConv [30]. Table 6 shows the comparison of Mean-per-IoU (mIoU) of FPConv [30] and GRA [28] evaluated on S3DIS dataset.

### 2.5. nuScene

The nuTonomy scenes (nuScenes) [18] collection is the first to include the whole autonomous vehicle sensor suite: six cameras, five radars, and one lidar, all with a full 360-degree field of view. nuScenes is made up of 1000 scenes, each of which is 20 s long and completely annotated with 3D bounding boxes for 23 classes and 8 characteristics. It has 7 times as many annotations and 100 times as many photos as the original KITTI dataset. nuScenes offers a significant advancement in terms of data quantities and complexity, and it is the first dataset to give 360-degree sensor coverage throughout the complete sensor suite. It is also the first AV dataset to incorporate radar data and was obtained using a public-road-approved AV. Furthermore, it is the first multimodal dataset to include data from dark and wet situations, as well as object features and scene descriptions in addition to object class and position. nuScenes is an AV standard for comprehensive scene knowledge. It enables the study of a variety of tasks such as object identification, tracking, and behaviour modelling in a variety of environments. The following methods discussed in this survey have validated their performances on this dataset: SLidR [7] and 3DSSD [23]. It was difficult to compare these models as the results published in the original paper were with different metrics.

### 2.6. ScanNet

ScanNet [19] is a dataset of richly-annotated RGB-D scans of real-world environments containing 2.5M RGB-D images for 1513 scans acquired in 707 distinct spaces. The extent of this research is largely due to its annotation with estimated calibration parameters, camera poses, 3D surface reconstructions, textured meshes, dense object-level semantic segmentations, and aligned CAD models. To design a framework that allows many people to collect and annotate large, a capture pipeline to make it easier for beginners to get semantically-labeled 3D models of scenes is constructed. RGB-D video is acquired and the data is processed offline. A complete semantically-labeled 3D reconstruction of the scene is returned. 3D deep networks can be trained with the data provided by ScanNet and their efficiency on many scene understanding tasks, including 3D object classification, semantic voxel labeling, and CAD model retrieval can be evaluated. There are several different types of locations in ScanNet, such as offices, housing, and restrooms. ScanNet provides a flexible framework for RGB-D acquisition and semantic annotations. ScanNet’s fully annotated scan data is helpful in achieving cutting-edge performance on a variety of 3D scene interpretation tasks. Finally, for reconstruction, instance-level object category annotations and 3D CAD model alignments are obtained from crowdsourcing using semantic annotation tasks. The following methods discussed in this survey have validated their performances on this dataset: GRA [28].

### 2.7. ScanObjectNN

ScanObjectNN [20] a point cloud object generated dataset using scene mesh data obtained from SceneNN [35] and ScanNet [19]. 700 distinct scenes are chosen from a total of more than 1600 scenes from SceneNN and ScanNet. To create a category for training data, each object is carefully reviewed, its inconsistent labels are rectified, and any confusing, poorly reconstructed, unlabeled, sparse, or small-instance objects are removed. Around 15,000 objects for 15 common categories are selected by design and the dataset is further enhanced by taking other object perturbations into account. Real-world objects were used to construct this dataset, which has greater benefits than utilizing artificial or synthetic datasets for learning. Classification models apply well to data from the actual world, such as point clouds created from RGB-D scans. In-context and comprehensive observations of actual objects are included in this collection. Models developed using this dataset are capable of handling background well when it coexists with objects due to clutter in real-world scenarios. This dataset provides additional real-world difficulties, such as background occurrence, object partiality, and many deformation variants. The following methods discussed in this survey have validated their performances on this dataset: GLR [31] and GDANet [33]. Table 7 shows the comparison of the accuracy of GLR [31] and GDANet [33] evaluated on this dataset.

## 3. Segmentation

3D object segmentation has applications in the fields of robotics, augmented reality, and medical picture analysis. It has received a lot of attention from the communities of computer vision, graphics, and machine learning. In this literature, numerous deep learning techniques for 3D semantic segmentation have been put forth which can be categorized into five groups based on: RGB-D images, projected images, voxels, points, and other representations. Point-based techniques can be further divided into multiple-layer perceptron (MLP), point convolution, and graph convolution techniques depending on the network design. Table 8 lists out the methods and Table 9 lists out their advantages and limitations that will be discussed in this section. SemanticKITTI [11] is one of the most common benchmarking datasets that many 3D segmentation methods use for evaluating their performances.

### 3.1. Bird’s Eye View (BEV) Projection Segmentation

**Figure 2:**
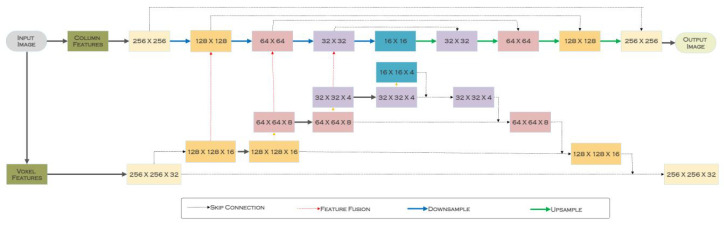
Network Architecture of 3D CNN model combining semantic segmentation and scene completion [25].

By conducting a point-level analysis for urban-size point clouds and presenting a multi-modal fusion segmentation model with a special Bird’s Eye View (BEV) [39] projection algorithm, the problem of 3D segmentation at the urban scale is effectively handled. A point level analysis is performed prior to model construction by projecting 3D points onto the BEV map and calculating the overlap ratio. To take advantage of both the 2D and 3D convolutional neural network, a combination of semantic segmentation and scene completion [25] is introduced. A 2D completion branch and an assisted 3D segmentation branch are two of the network’s components. Since 3D dense convolution uses too many resources and 3D sparse convolution makes it challenging to create unique voxels, the scene is completed using a 2D network using BEV. Additionally, distributing features with 2D convolution is effective and simple. In order to combine the benefits of 2D and 3D networks from multi-view fusion, the attributes of the semantic segmentation branch are included as an auxiliary. These features can continually provide semantic features to the completion branch. The foundation of the system is a 2D encoder-decoder architecture since 2D networks are lighter and more practical for distributing features. Instead of the conventional spherical projection, Cartesian voxelization is implemented in segmentation. To create a BEV feature map, a top-down approach is used. The authors evaluated the performance of this model on SemanticKITTI [11] dataset. This model was able to achieve a mean IoU of 58.8 and performed comparatively better. Figure 2 shows the network architecture of this 3D CNN model where the lower part of the figure is an auxiliary 3D semantic segmentation branch, and the upper part is a 2D completion branch that follows the UNet structure and performs four downsamplings.

This method demonstrates that point cloud completion and semantic segmentation may be performed concurrently by exchanging semantic and geometrical information. This work uses extra semantic-related input to accomplish more realistic scene completion by accepting semantics as inputs and demonstrates that 3D partial observations and semantic information are complimentary to one other by displaying amazing results [40]. This work used BEV semantic map as a scene completion task to inpaint sparse semantic LiDAR points into semantic map [41]. Occlusion is considered to be one of the key challenges when implementing change detection on 3D point clouds. During Occlusion, point clouds appear incomplete, that is, the point clouds will appear on one scan but not in the other. This paper has addressed this issue of Occlusion by using deep learning to fill in the occluded parts [42]. This approach depends heavily on voxel-wise completion labels and performs poorly on little, distant objects and cluttered scenes [43].

### 3.2. Group Relation Aggregator (GRA)

**Figure 3:**
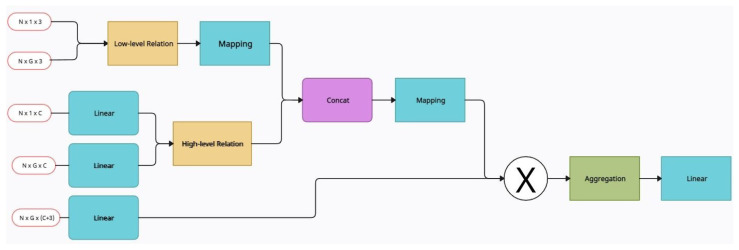
An overview of Group Relation Aggregator (GRA) [28].

When compared to self-attention and set-abstraction techniques, the Group Relation Aggregator (GRA) [28], which is proposed to learn from both low-level and high-level relations, and is efficient in terms of computation and the number of parameters. The structural and semantic correlations between points are encoded by this scalable local aggregator for point clouds. Point-based network RPNet is built by utilizing bottleneck GRA. The bottleneck is constructed by taking the performance of GRA into account. RPNet with reference to width (RPNet-W) and depth (RPNet-D) are developed based on this recommended module. RPNet is a flexible and highly productive hierarchy. This expansive RPNet greatly boosts efficiency when configured with the bottleneck version of the aggregator. Only GRA is utilized by RPNet-W, which is used for categorization. For segmentation tasks, however, RPNet-D with skip block (GRA with down-sampling) and residual block (GRA with a residual link) are implemented. The model is evaluated on the classification dataset ModelNet40 [17] and segmentation datasets, ScanNet [19] and S3DIS [12]. The performance of the model is compared to FPConv [30]. The model outperformed FPConv [30]. The model was able to achieve an accuracy of 94.1 on ModelNet40 [17], 70.8 on S3DIS [12], and 68.4 on ScanNet [19]. Whereas FPConv [30] was able to achieve an accuracy of 92.5 on ModelNet40 [17], 68.7 on S3DIS [12] and 63.9 on ScanNet [19]. An overview of GRA can be observed in Figure 3.

This approach is theoretically simpler and provides results comparable to, if not better than, several cutting-edge methods [44]. Local shapes are essential for learning point clouds. This approach uses relations to learn from local structural information [45]. RPNet is not convolutional as the input of its MLPs contains the absolute location of the points, unlike other point-convolutional layers which incorporate the relative position of the points with respect to the output [46]. This method employs a technique borrowed from "learn from relation" by first encoding local coordinate information to mitigate the sparsity and multi-scale issues of large-scale point cloud images [47].

### 3.3. HiLo-Network

HiLo-Network [21] a 3D semantic segmentation method is introduced as HiLoNetworks can be applied to a broader range of 3D datasets. The main goal of this model is to reduce memory consumption while retaining a fast training process. Multiple forward passes are traded-off at inference time to obtain a scalable approach that can run on most consumer-level GPUs. This method is specifically developed to be used for commercial detection purposes by limiting their production costs. HiLo-Network overcomes the challenge of a super-resolution deep neural network in retrieving high-resolution information from low-resolution representations. A divide-and-conquer procedure to semantic segmentation is applied to improve the GPU acceleration of gradient-based optimization. Due to this, during gradient descent, only small chunks (a window) of each instance within each batch will be loaded into Video RAM instead of passing a complete volume into a network. To overcome the problem of global relations between different windows not being taken into consideration, a second window (centered around the first window) is constructed and down-sampled using average pooling. The performance of this model is evaluated by the 3D-CT dataset, artificially created with a limited number of objects. The model was able to acquire an IoU of 0.6838.

Two innovative designs for 3D semantic segmentation of voxelized volumes are suggested in this paper. For weapon detection in baggage, the approaches are evaluated using a 3D CT scan dataset. The introduction of a high-resolution Occupancy Network. Sadly, none of the evaluated super-resolution O-Net topologies can attain the necessary results. HiLo-Network, a new scaleable neural network architecture for 3D semantic segmentation, is suggested. HiLo-Networks can successfully separate firearms within baggage, according to this article. They are memory efficient and scalable in terms of input resolution. HiLo-Networks, in particular, may be trained on consumer-level GPUs.

### 3.4. Swin UNETR

**Figure 4:**
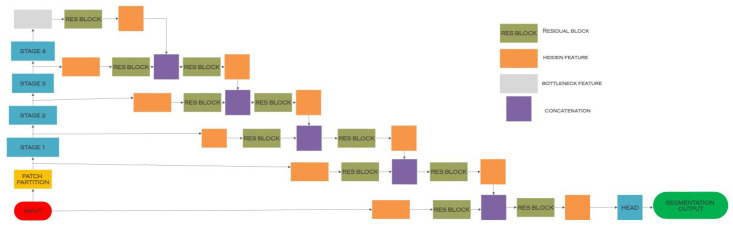
Overview of the Swin UNETR architecture is observed [37].

Swin UNETR [37] utilizing a U-shaped network with a Swin transformer as the encoder and connecting it to a CNN-based decoder at different resolutions via skip connections is introduced for semantic segmentation of brain tumors using multi-modal MRI images. Swin transformers are suitable for various downstream tasks wherein the multi-scale features extracted can be leveraged for processing. The model takes 3D multi-modal MRI images with 4 channels as input. The Swin UNETR creates non-overlapping patches of the input data by using a patch partition layer to create windows with a desired size for computing the self-attention. The encoded feature representations in the Swin transformer are then fed to the CNN-decoder via skip connection at multiple resolutions. Final segmentation output containing 3 output channels corresponding to Whole Tumor (WT), Tumor Core (TC) and Enhancing Tumor (ET) sub-regions are used. The superior performance of the Swin UNETR model for brain tumor segmentation is mainly due to its capability of learning multi-scale contextual information in its hierarchical encoder through the self-attention modules and effective modeling of the long-range dependencies. This model is trained and evaluated on the BraTs 2021 [38] which contains 1251 participants, each with four 3D MRI modalities. The annotations of this dataset were divided into three sub-regions: Whole Tumor (WT), Tumor Core (TC) and Enhancing Tumor (ET). The performance of this model is evaluated in the form of a Dice Score and the model was able to achieve a Dice Score of 0.927. Figure 4 shows the overview of Swin UNETR architecture.

Swin UNETR computes self-attention via an efficient shifting window partitioning algorithm and ranks first on the BraTs 2021 [38] validation set [48]. This approach, which is commonly used in medical imaging applications, is built on top of a SWin Transformer to extract and down-sample feature maps before feeding them into a Transformer [49]. This model performs segmentation of tumor pixels with 0.92 dice similarity coefficient [50]. While transformers have been used successfully in computer vision applications, this technique investigated the use of transformers in medical image processing by replacing the convolutional encoding and decoding procedures in U-Net with a Swin Transformer module and establishing Swin-UNet [51].

### 3.5. Meta-RangeSeg

**Figure 5:**
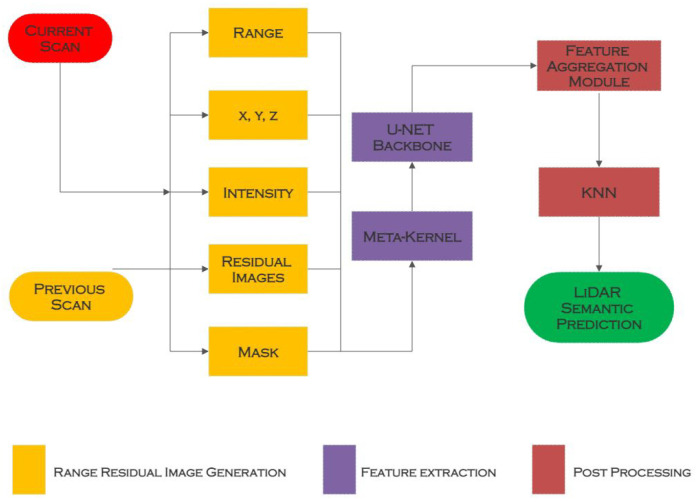
An overview of Meta-RangeSeg framework [15].

Meta-RangeSeg [15], an approach to semantic segmentation for LiDAR sequences is proposed, where a range residual image representation is introduced to capture the spatial-temporal information by employing Meta-Kernel to extract the meta-features and reduce the inconsistency between the 2D range image coordinates input and Cartesian coordinates output. This channel takes advantage of the range residual image with nine channels built from scans. Meta features are extracted by the Meta-Kernel block, and multi-scale features are obtained via the U-Net network. Final labels for raw data are obtained by post-processing the aggregated features. Feature Aggregation Module (FAM) aggregating the meta-features and multi-scale features to strengthen the role of the range channel at various scaled for object segmentation is also introduced. Range image representation is introduced into the task of semantic segmentation on LiDAR sequences to capture the temporal information as it has the advantage of effective 2D operations for fast training and inference. Performance of the model is evaluated with SemanticKITTI [11] dataset. The model was able to get a mean IoU of 0.537. Figure 5 shows the overview of the Meta-RangeSeg framework where the range residual images are generated, and meta-features are extracted and aggregated to produce semantic labels in 3D space.

This technology is developed to assist individuals in creating after-effects creation, background music production, video dubbing, and other postproduction as deep learning continues to improve in the video and audio domains [52]. The boundary loss function is employed in this technique for LiDAR semantic segmentation to account for the problem of hazy segmentation borders [53].

### 3.6. SLidR

**Figure 6:**
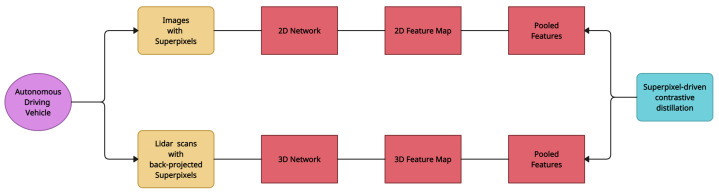
Superpixels used in SLidR can be observed [7].

SLidR [7] is a self-supervised method for tasks such as semantic segmentation or object detection in Lidar point clouds, and is designed to be tailored to autonomous driving data. Autonomous driving vehicles equipped with an array of cameras and Lidar sensors, offer rich surround-view information which is leveraged to distill self-supervised pre-trained image representations into a 3D network. This pre-training process does not require any annotation of the images or of the point clouds. This method has also shown that self-supervised pre-training on images for learning generic representations can also be used to pre-train 3D networks for autonomous driving. This self-supervised 2D-to-3D representation distillation approach is based on a superpixel-to-superpoint contrastive loss and a carefully designed image feature upsampling architecture which allows high-resolution image features to be distilled without suffering from degenerate solutions. This method also provides the study on the self-supervised image-to-Lidar representation distillation problem for autonomous driving data. As shown in Figure 6, SLidR distillates the knowledge of a pre-trained and fixed 2D network into a 3D network using superpixels to pool features of visually similar regions together, both on the images and on the point clouds through superpixels back-projection. The model is evaluated on nuScenes [18] and KITTI 3D object dataset [16]. SLiDR was able to achieve a mean average precision (mAP) of 74.6 on nuScenes [18] and 62.4 on KITTI 3D object dataset [16].

This approach for using knowledge distillation for 3D detection has been proposed. This approach used matched real-world 2D-3D data of outdoor settings and contrastive learning to transmit information [54]. Nevertheless, in a multi-modal situation, this technique concentrates on the selection of student-teacher, such as educating point clouds-based student detectors with an images-based instructor or vice versa, while ignoring the distinctive qualities of point clouds. Also, the construction of specific knowledge distillation optimisation algorithms for point cloud-based pure 3D detection has not been thoroughly investigated [55]. This method is heavily reliant on a huge collection of annotated point clouds, which is especially important when high-quality 3D annotations are expensive to get [56].

## 4. Object Detection

Object Detection is actively researched as many practical applications utilise these to locate the relevant objects in the given scene. Point clouds pose some additional complexity over image object detection models and this requires a further need for optimisation. Some of the object detection methods include discretization-based methods, point-based methods, and multi-view methods. Discretization-based detection techniques are based on applying random sampling to the points within each of the voxels and passing them through feature encoding layers. These methods invariably lose spatial information and are unable to fully utilize 3D point cloud structural information, which reduces the accuracy of their localization. Point-based methods often try to minimise the spatial information loss while extracting the features and therefore mostly outperform the other downscaling-based and multi-view methods. Multi-view methods often fuse proposal-wise features from different view maps and their computation cost is higher than the other methods. Table 10 lists out the methods and Table 11 lists out their advantages and limitations that will be discussed in this section.

### 4.1. VoxelNet

**Figure 7:**
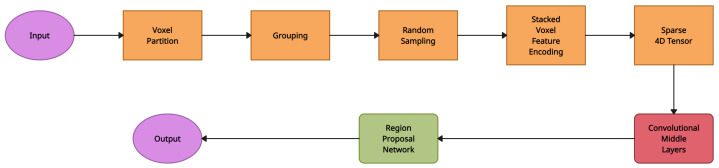
An overview of VoxelNet Architecture [1].

As CNN demonstrated promising results in image object detection, this often inspired the application of 3D CNNs on the projected point clouds. VoxelNet [1] is one of the recent approaches which applies random sampling to the points within each of the voxels and passes them through feature encoding layers. The extracted features are later on used by a region proposal network (RPN) to produce object detection results. The RPN is a highly optimised object detecting system. This strategy, however, necessitates dense data arranged in a tensor form (e.g., picture, video), which is not the case for ordinary LiDAR point clouds. As expected from 3D representations, VoxelNet is relatively slow due to the sparsity of the input data and 3D convolutions. Figure 7 shows the overview of VoxelNet Architecture, where the feature learning network takes a raw point cloud as input, partitions the space into voxels, and transforms points within each voxel to a vector representation characterizing the shape information. VoxelNet is evaluated on KITTI 3D object dataset [16] and was able to achieve an average precision of 81.97.

VoxelNet uses a PointNet [57] within each voxel to provide a uniform feature representation from which a head employing 3D sparse convolutions and 2D convolutions generates detections. A 3D encoder is used in this approach to quantizing the point cloud into regular bins [58]. This grid-based technique often converts irregular point clouds to regular representations such as 3D voxels, which may then be processed effectively by 3D or 2D Convolutional Neural Networks (CNN) to learn point characteristics for 3D detection. It separates point clouds into 3D voxels for 3D CNN processing, and it introduces 3D sparse convolution for efficient 3D voxel processing [59]. This approach demonstrates that switching from a box representation to a center-based representation results in a 3–4 mAP boost in 3D detection [58]. Because of the computational expense, the input 3D grid is limited to low resolution, resulting in structural information loss [28].

### 4.2. Sparsely Embedded CONvolutional Detection (SECOND)

**Figure 8:**
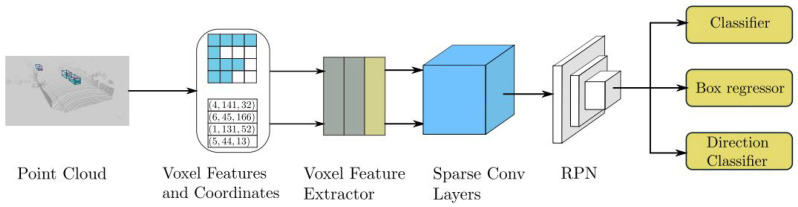
The Structure of SECOND detector [2].

The computational expense of VoxelNet [1] is one of its main drawbacks, making it challenging to apply for real-time applications. A successor network called SECOND (Sparsely Embedded CONvolutional Detection) [2], which makes the most of the rich 3D information inherent in point cloud data, has been presented as a solution to this problem. The convolutional network design is enhanced by this technology in numerous ways. In order to obtain information from the z-axis before the 3D data are downscaled to resemble 2D picture data, spatially sparse convolutional networks are introduced for LiDAR-based detection. A rule generation technique for sparse convolution to increase speed which is GPU (Graphical Processing Unit) based is introduced. Applying direct transformations to specific points on an object using point cloud data makes it incredibly simple to scale, rotate and move the object. Based on this feature, SECOND incorporates a unique type of data augmentation. The properties of objects and the related point cloud data are created in a ground-truth database. During training, objects extracted from this database are subsequently added to the point clouds. This strategy has the potential to significantly improve our network’s ultimate performance and convergence speed. In order to address the issue of the significant loss created when the difference in orientation between the ground truth and the prediction is equal to π, a unique angle loss regression technique is developed. This strategy produces a bounding box that is similar to the actual bounding box. SECOND is evaluated on KITTI 3D object dataset [16] and the performance of the model is compared with AVOD [9]. SECOND was able to achieve an average precision of 83.13 and AVOD scored 73.59. SECOND outperformed AVOD. Figure 8 shows the structure of the SECOND detector.

This approach, similar to VoxelNet [1], demonstrates that switching from a box representation to a center-based representation results in a 3-4 mAP boost in 3D detection. SECOND streamlines the VoxelNet and accelerates sparse 3D convolutions. Similar to VoxelNet [1], a 3D encoder is used in this approach to quantizing the point cloud into regular bins. This anchor-based 3D detector relies on 2D Box IoU for target assignment during training, which adds needless costs when selecting positive/negative thresholds for different classes or datasets [58]. Like VoxelNet [1], This grid-based technique often converts irregular point clouds to regular representations such as 3D voxels, which may then be processed effectively by 3D or 2D Convolutional Neural Networks (CNN) to learn point characteristics for 3D detection. It separates point clouds into 3D voxels for 3D CNN processing, and it introduces 3D sparse convolution for efficient 3D voxel processing [59]. SECOND increased VoxelNet [1] inference performance, but 3D convolutions remain a problem.

### 4.3. PointPillars

**Figure 9:**
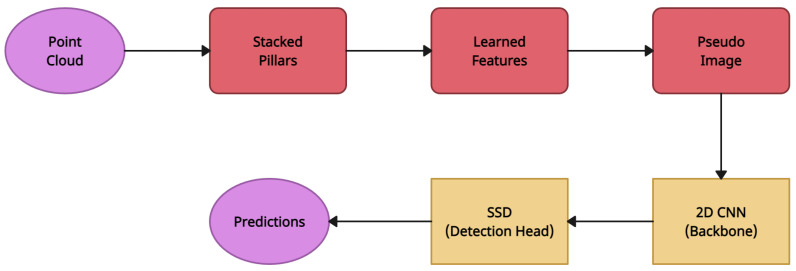
The Network Overview of PointPillars [5].

A 3D object identification technique called PointPillars [5] allows for end-to-end learning with only 2D convolutional layers. In order to predict 3D-oriented boxes for objects, PointPillars use a new encoder that learns features on the pillars (vertical columns) of the point cloud. This technique has a variety of advantages. First, PointPillars will make use of the complete information provided by the point cloud by learning features rather than depending on fixed encoders. Secondly, as pillars are used instead of voxels, manual vertical direction binning optimization is not required. Finally, pillars are fast and accurate due to the fact that all critical operations can be expressed as 2D convolutions, which are highly efficient to compute on a GPU. PointPillars does not require manual adjustment to employ various point cloud configurations, including multiple LiDAR scans or even radar point clouds, which is another advantage of learning features. This model is evaluated on KITTI 3D object dataset [16] and the performance is compared with VoxelNet [1] and SECOND [2]. PointPillars was able to obtain the mean average precision of 66.19. Whereas VoxelNet [1] and SECOND [2] got the mean average precision of 58.25 and 60.56, respectively. Figure 9 shows the main components of the PointPillar network which includes Pillar Feature Network, Backbone, and Single-Shot Detector (SSD) Detection Head.

PointPillars model is created to construct a 3D Item Detection baseline, which employs a single layer PointNet [57] to voxelize the point cloud into the Birds Eye View, followed by a CNN area proposal network [60]. Given the success of 2D CNNs, this method employs multi-view projection, in which 3D point clouds are projected into several picture planes. Next, in these picture planes, 2D CNNs are utilised to extract feature representations, which are then fused with multi-view feature representations to generate the final output representations [61]. Lidar point clouds provide less semantic information but provide very accurate 3D localisation as the reflectance of lidar is an essential feature. PointPillars demonstrated a lidar-only solution that outperformed previous fusion-based algorithms. This implies that further effort is needed to integrate multimodal measures in a principled way. This approach is thought to be the quickest recorded method in terms of inference time [18].

### 4.4. Structure-Aware Single Stage 3D Object Detector (SA-SSD)

**Figure 10:**
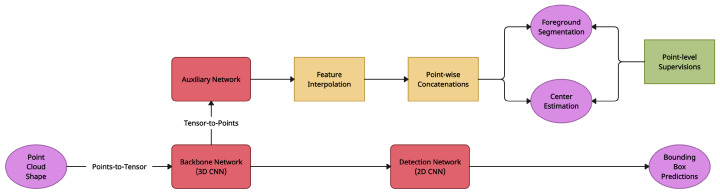
The Network Overview of structure aware single-stage 3D object detector [6].

A single-stage 3D object detector [6] that is aware of structure is created to make use of fine-grained structure information to increase localization accuracy while maintaining the high efficiency of single-stage techniques. In the detector, which is depicted in Figure 10, there is a backbone network that produces downscaled features for bounding box prediction and an auxiliary network that directs the backbone network to learn additional discriminative features using point-level supervisions. In order to make the features more sensitive to object boundaries and aware of intra-object relationships, the auxiliary network first converts the features from the backbone network back to point-wise representations. It then performs two auxiliary tasks: foreground segmentation and point-wise center estimation. By performing a spatial transformation on the classification feature maps, an effective part-sensitive warping method can be used to align the classification confidences with the predicted bounding boxes, improving the model’s capability of producing reliable confidence maps. Figure 10 shows the overview of the network architecture of the structure aware single-stage 3D object detector. This model is evaluated on KITTI 3D object dataset [16] and the performance is compared with VoxelNet [1], SECOND [2] and PointPillars [5]. SA-SSD was able to obtain the mean average precision of 88.75. Whereas VoxelNet [1], SECOND [2], and PointPillars [5] got the mean average precision as 77.82, 83.34 and 82.58, respectively. Compared to SECOND [2], using a voxel-free encoding pre-process can help to save up to 6.6 ms.

Similar to VoxelNet [1] and SECOND [2], a 3D encoder is used in this approach to quantizing the point cloud into regular bins. This approach aggregates grid point features from three nearby non-empty 3D feature volumes using a radial basis function [58]. To maintain structural information, this method presents an auxiliary network and losses based on SECOND [2,62]. This work tries to enhance feature representation by utilising auxiliary tasks or extra constraints without incurring additional computing burdens during inference. To augment the features, this approach uses an auxiliary network in parallel with the backbone to regress box centres and semantic classes. This method employs a lightweight BEV network for robust spatial-semantic feature extraction, together with IoU-aware confidence correction for improved post-processing [63].

### 4.5. Sparse-to-Dense 3D Object Detector (STD)

**Figure 11:**
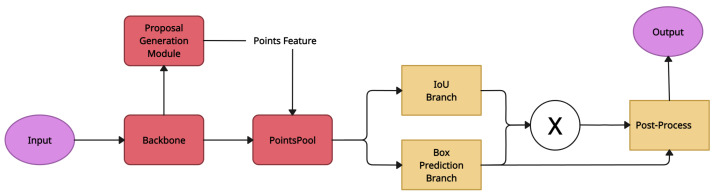
The Network Overview of STD Framework [4].

A two-stage STD architecture [4] for 3D object detection is developed. To preserve precise position information, each point in the point cloud is treated as an element in the first stage and seeded with the necessary spherical anchors. Then, using a PointNet++ [36] backbone, semantic context features are extracted for each point and an object class score is generated to filter anchors. In order to produce features for each proposal, a PointsPool layer is proposed by compiling the canonical coordinates and semantic characteristics of the inner points while maintaining precise localization and context data. The use of effective CNNs and end-to-end training is made possible by this layer, which converts sparse and unordered point-wise expressions into more compact features. In order to prevent incorrect removal during post-processing, a 3D IoU branch is added to the prediction of 3D IoU between predictions and ground-truth bounding boxes. This model is evaluated on KITTI 3D object dataset [16] and the performance is compared with AVOD [9], VoxelNet [1], SECOND [2] and PointPillars [5]. SA-SSD was able to obtain the mean average precision of 86.61. Whereas AVOD [9], VoxelNet [1], SECOND [2] and PointPillars [5] got the mean average precision as 73.59, 77.47, 83.13 and 79.05, respectively. Figure 11 shows the overview of the STD framework which contains a Proposal Generation Module (PGM), PointsPool layer, and a box prediction network.

This segment-based network employs point-wise feature extraction recursively. To enhance inference time, a single classifier is learned on the Encoding-Decoding Feature Pyramid in this technique. STD is a hybrid detector that relies on both anchors and point masks to generate region proposals. STD naturally takes RoI characteristics from RPNs and then optimises the imperfect bounding box proposals from earlier stages by predicting and fixing residual size and placement (centre and orientation) relative to the input bounding box predictions. STD is a Prediction Refinement Subnetwork that promotes prediction refinement independence. STD introduces a refinement network that is completely independent of the previous pipeline step, which increases inference time but provides more alternatives in terms of training and testing methodologies, resulting in better results. STD uses a Point-based data structure with a Mask-level detection option, which implies that bounding box suggestions are created directly from the segmented foreground points [64]. Without upsampling, and simply detecting on remaining downsampled points in STD, performance lowers by around 9 percent [23].

### 4.6. PointRCNN

**Figure 12:**
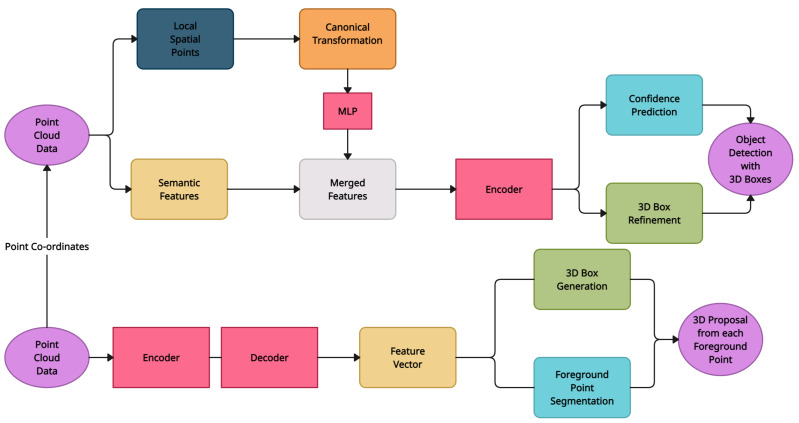
The Network Architecture of PointRCNN [3].

PointRCNN [3], a two-stage framework for 3D object identification, is created. It works directly with 3D point clouds to produce reliable and precise 3D detection results. The proposed framework is divided into two stages, the first of which tries to provide a bottom-up proposal for a 3D bounding box. The first stage divides the foreground points and produces a limited number of bounding box suggestions to construct the ground-truth segmentation mask. This technique eliminates the need for a huge number of 3D anchor boxes throughout the whole 3D space, such as VoxelNet [1] does and saves a significant amount of processing. Canonical 3D box refining is carried out by PointRCNN’s second step. A point cloud region pooling procedure is employed to pool stage-1 learnt point representations once the 3D proposals have been created. For learning relative coordinate refinement, the pooled 3D points are translated to canonical coordinates and coupled with the pooled point features and the stage-1 segmentation mask. VoxelNet [1] can also be adopted as a backbone for this network instead of PointNet++ [36]. This model is evaluated on KITTI 3D object dataset [16] and the performance is compared with VoxelNet [1] and SECOND [2]. PointRCNN was able to obtain the mean average precision of 88.88. Whereas VoxelNet [1] and SECOND [2] got the mean average precision of 81.98 and 87.43, respectively. As shown in Figure 12, the network consists of parts for generating 3D proposals from the raw point cloud in a bottom-up manner and for refining the 3D proposals in canonical coordinates.

For 3D detection, this point-based technique extracts discriminative features directly from raw point clouds. In general, grid-based algorithms are more computationally efficient, but the unavoidable information loss reduces the accuracy of fine-grained localisation. The point-based approaches, on the other hand, have a greater computing cost but can easily obtain a bigger receptive field due to the point-set abstraction. For 3D detection using point clouds alone, PointRCNN creates 3D suggestions straight from the entire point cloud instead of 2D pictures [59]. AB3D [65] developed over this model combines a Kalman filter with precise 3D detections to deliver cutting-edge performance [66]. PointRCNN generates proposals using the entire point cloud rather than 2D pictures. It immediately employs the proposal’s focal point segmentation score for categorization while taking proposal location information into account. Other characteristics, such as size and direction, are overlooked [4].

### 4.7. 3DSSD

**Figure 13:**
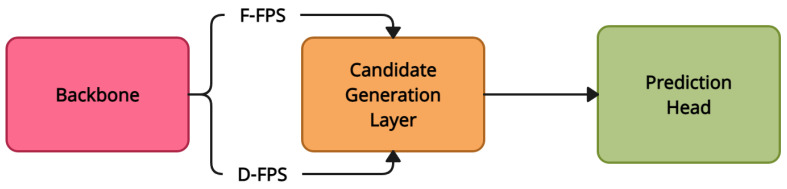
An Overview of 3DSSD Framework [23].

A box prediction network called 3DSSD [23] is designed to more effectively use the representative points preserved after Set Abstraction (SA) layers. This makes use of a 3D center-ness assignment approach, an anchor-free regression head, and a candidate generation layer (CG). To create candidate points in the CG layer, a representative sampling strategy based on feature distance (F-FPS) points are shifted. The distances between the representative points and the centers of their respective instances serve as the regulators of this shifting process. Multi-layer perceptron (MLP) networks are used to extract the features of these candidate points’ surrounding points, which are retrieved from the whole set of representative points from both the F-FPS and the furthest-point-sampling based on 3D Euclidean distance (D-FPS). These candidate points are then considered centers. In order to predict 3D bounding boxes, these characteristics are eventually loaded into an anchor-free regression head. In order to obtain accurate localization prediction, a 3D center-ness assignment technique is designed that gives better classification scores to candidate points that are closer to instance centers. Performance of this model is evaluated on KITTI 3D object dataset [16] and is compared with VoxelNet [1] and SECOND [2]. 3DSSD was able to obtain the mean average precision of 89.71. Whereas VoxelNet [1] and SECOND [2] got the mean average precision of 81.98 and 87.43, respectively. Similarly, the performance of this model is evaluated on nuScenes [18] and is compared with SECOND [2] and PointPillars [5]. 3DSSD was able to obtain the mean average precision of 81.20. Whereas SECOND [2] and PointPillars [5] got the mean average precision of 75.53 and 70.5, respectively. Figure 13 shows the overview of this framework which consists of a backbone network, a Candidate Generation (CG) layer, and an anchor-free prediction head.

This point-based 3D single-stage object detector, which includes a fusion sampling approach in the downsampling process, a candidate generation layer, and an anchor-free regression head with a 3D center-ness assignment technique, achieves a good combination of accuracy and efficiency [67]. This technique is a point-based strategy that acts directly on point clouds and creates 3D bounding boxes. These methods, which mainly use point operators to extract features directly from point clouds, suffer from the sparse and non-uniform point distribution, as well as the time-consuming process of sampling and searching for nearby points [68].

### 4.8. IMVoteNet

**Figure 14:**
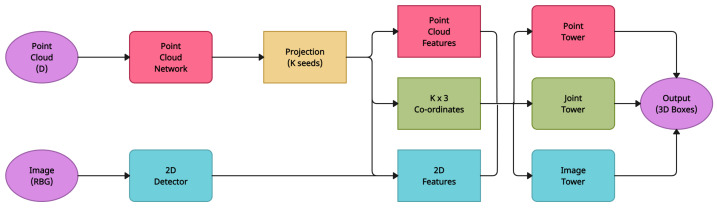
An Overview of the 3D object detection pipeline for IMVoteNet [13].

VoteNet [69] is a point cloud-focused 3D detection framework that analyzes raw data directly and doesn’t rely on any 2D detectors, either in terms of design or object proposal. This network draws its inspiration from the extended Hough voting method for object recognition and is based on recent developments in 3D deep learning models for point clouds [70]. To reduce the requirement of converting point clouds to normal structures, PointNet++ [36], a hierarchical deep network, is used. A voting method is built into point cloud deep networks to create new points at the centers of objects, which may then be combined and aggregated to produce box suggestions. A hybrid 2D-3D voting technique for 3D object recognition called IMVoteNet [13] is created based on the VoteNet architecture and design to make use of geometric and semantic/texture signals in 2D pictures. Instead of depending entirely on 2D detection, "pseudo" 3D votes are created by lifting 2D votes from the picture space and converting them to 3D using geometric transformations based on camera intrinsics and pixel depth. These pseudo-3D votes are added as additional features to the 3D seed points for object proposals. These features are concatenated with the 3D point features from a point cloud backbone network after lifting and converting all the features from the pictures to 3D. The information from the two modalities can be properly balanced by combining 2D and 3D sources. To make the most of both the 2D and 3D characteristics, a multi-towered network structure with gradient mixing is employed. This model is evaluated on SUN RGB-D [71] dataset which is a single-view RGB-D dataset for 3D scene understanding. It comprises around 10K RGB-D photos, with approximately 5K for training. Each image is labelled with 3D modal bounding boxes. A total of 37 item categories have been annotated. The model was able to acquire a mean average precision (mAP) of 58.6. As shown in Figure 14, the model initially has two separate branches for 2D object detection and point cloud feature extraction, which are then lifted and fused to generate votes towards 3D object centers and propose 3D bounding boxes with its features in the joint tower.

Instead of immediately regressing to 3D bounding boxes using features at the centre point, this technique identifies objects by vote clustering utilising point feature sampling and grouping [58]. This point-based technique is primarily based on the PointNet series, particularly the set abstraction operation, which permits adjustable receptive fields for learning point cloud features [59]. This method serves as a foundation for many subsequent projects. While effective, this approach took years to perfect by hand-encoding inductive biases, radii, and constructing specific 3D operators and loss functions. Because the loss employed in VoteNet does not prevent numerous predictions of the same item, it depends on Non-Maximal Suppression as a post-processing step to eliminate them [72].

### 4.9. Aggregate View Object Detection (AVOD)

**Figure 15:**
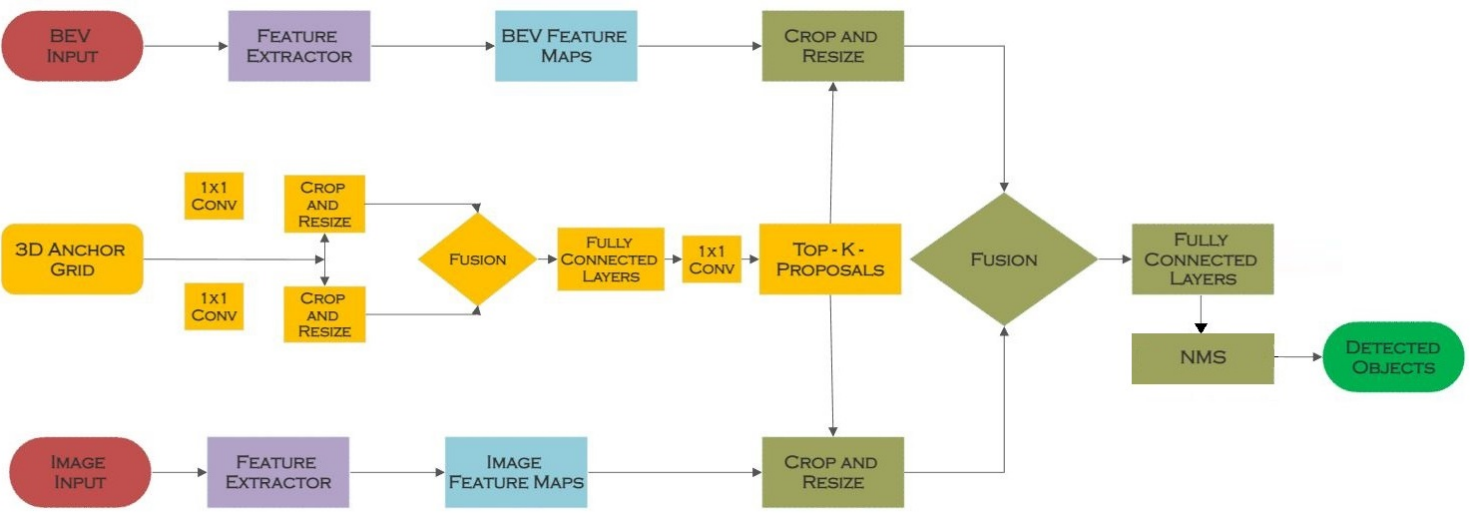
Network Architecture of AVOD [9].

A feature fusion region proposal network (RPN) and a distinctive 3D bounding box encoding form the Aggregate View Object Detection (AVOD) [9] architecture for autonomous driving. The localisation of smaller classes in the scene is made possible by the feature extractor, which creates high-resolution feature maps from LIDAR point clouds and RGB pictures. For small classes, the feature fusion RPN generates high recall region recommendations by combining different modalities. Higher 3D localization accuracy is achieved by the 3D bounding box encoding’s adherence to box geometric restrictions. The neural network architecture takes advantage of 11 convolutions at the RPN stage to preserve detection performance while enabling fast computational speed and a small memory footprint. This network is made a viable contender for deployment on autonomous cars by being incorporated into the autonomous driving stack. Performance of this model is evaluated on KITTI 3D object dataset [16] and is compared with VoxelNet [1]. AVOD was able to obtain the mean average precision of 81.94. Whereas VoxelNet [1] got the mean average precision of 77.47. In Figure 15, the feature extractors are shown in pink, RPN in blue, and the second stage detection network in green.

This grid-based technique converts irregular point clouds to regular representations such as 2D bird-view maps, which may then be effectively processed by 3D or 2D Convolutional Neural Networks (CNN) to train point features for 3D detection [59]. When performing object detection from point clouds, there are two fundamental differences: (1) A point cloud is a sparse representation, whereas an image is dense; and (2) A point cloud is 3D, whereas an image is 2D. As a result, object recognition from point clouds is not easily accomplished using traditional image convolutional processes. This approach provides a birds-eye perspective of the lidar point cloud (BEV). Nevertheless, because the bird’s eye view is so sparse, the direct use of convolutional neural networks is impracticable and wasteful. To address this issue, this approach divides the ground plane into regular grid cells, such as 10 × 10 cm, and then applies a hand-crafted feature encoding algorithm to the points in each grid cell. Such solutions, however, may be suboptimal since the hard-coded feature extraction method may not extend to new setups without substantial engineering work [5]. AVOD combines lidar and image data to generate a multi-modal detector, necessitating the usage of two-stage detection pipelines [5]. AVOD still has a limitation when detecting small objects, such as pedestrians and cyclists as it does not deal with cases with multiple objects in depth direction [4].

### 4.10. FuDNN

**Figure 16:**
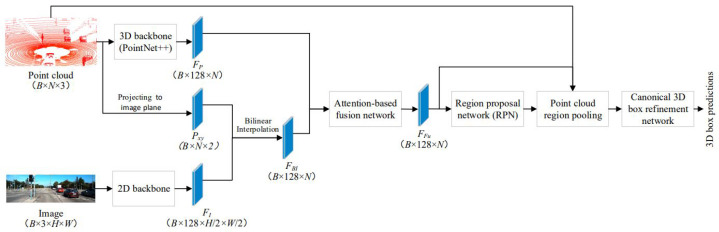
Network Architecture of FuDNN [24].

For LiDAR-camera fusion 3D object identification, a novel deep neural network called FuDNN [24] based on PointRCNN [3] is created. In order to learn 2D features from camera images, a 2D backbone is proposed. In order to improve results, an attention-based fusion sub-network is created to fuse the 2D (image features) and 3D (point cloud features) data collected from 3D LiDAR point clouds. Compared to other 2D backbones, the one presented in this network has a more compact structure yet performs better. The RPN and 3D box refinement network of PointRCNN [3] are used, respectively, to produce 3D proposals and improve the 3D box placements. Performance of this model is evaluated on KITTI 3D object dataset [16] and is compared with PointPillars [5], SECOND [2] and PointRCNN [3]. FuDNN was able to obtain the mean average precision of 92.48. Whereas PointPillars [5], SECOND [2] and PointRCNN [3] got the mean average precision as 87.75, 90.97 and 92.54, respectively. The architecture of FuDNN is shown in Figure 16, including a 2D backbone, a 3D backbone, an attention-based fusion sub-network, an RPN, and a 3D box refinement network.

FuDNN is an attention module-guided feature fusion model for LiDAR and camera data. This is a multi-view-based fusion model, a feature-level fusion model that creates 3D region proposals based on a bird’s-eye view and conducts 3D bounding box regression. The drawback with this technique is that when the point cloud data is sparse, the texture information in the picture data may not be properly exploited [73]. The RPNs utilised in this approach to build bounding boxes for object categorization and regression, are constrained by their significant latency.

## 5. Deep Learning Based 3D Object Classification

Predicting the class of a 3D object using its point cloud is known as 3D object classification. Each voxel is categorized into a category in this voxel-level prediction. In this literature, numerous deep learning techniques for 3D object classification have been put forth which can be categorized into different groups based on RGB-D images, projected images, voxels, points, graphs, and other representations. Table 12 lists out the methods and Table 13 lists out their advantages and limitations that will be discussed in this section.

### 5.1. OctNet

OctNet Octnet [10] is a 3D-convolutional network that divides the 3D space into imbalanced octrees in a hierarchy, with each octree dividing the space based on the density of the data. Depending on the 3D structure of the input, this network recursively separates octree nodes that contain data points in its domain and dynamically concentrate on computational and memory resources. As a result, the computational and memory requirements are significantly reduced, enabling deep learning at high resolutions. The maximum responses across all feature maps at various network layers are represented using this technique. For 3D classification, 3D orientation estimation of instances of unknown objects, and semantic segmentation of 3D point clouds, OctNet is recommended for use. Due to the low memory usage, this enables higher input resolutions, which are ideal for orientation estimation and semantic point cloud labeling. Performance of the model is evaluated on ModelNet10 [17] dataset. The model was able to achieve an accuracy of 81.5.

OctNet employs octrees that are imbalanced and have hierarchical divisions [61]. Utilizing sparse structures like octrees allows for wider grids and improved speed, however, this network lacks flexibility because its kernels are limited to $3^{3}$ = 27 or $5^{3}$ = 125 voxels [76]. The problem of non-uniform sample density has not been clearly addressed in this study [36]. An octree-based technique overcomes the computation and memory constraints of dense voxel methods, allowing for the capacity to learn at up to $512^{3}$ resolution, yet even this resolution is far from making visually appealing forms [77]. Because of the computational expense, the input 3D grid is limited to low resolution, resulting in structural information loss [28].

### 5.2. RotationNet

RotationNet [22] is a CNN model that predicts an object’s posture and object category using Multiview images of the object. For each image input, RotationNet produces category likelihoods that are view-point-specific and correspond to all preset discrete viewpoints. The selected object posture optimizes the category of the integrated object. RotationNet can be used to perform on-the-fly classification with a moving camera since it permits consecutive input and updates of the category probability of the target object. A complete collection of multi-view images of an object taken from each of the pre-defined views is used for training, and just a portion of the complete set is used for testing or inference. It does not require the images to be delivered all at once and permits sequential input of multi-view images. Using an unaligned object dataset enables unsupervised learning of object postures. The multi-view representation divides the three-dimensional volume into two-dimensional picture slices. As a consequence, AlexNet is used, but only for 2D convolution. Yet, some global context is lost as a result of the axis separation [21]. The model is evaluated on ModelNet10, ModelNet40 [17], and MIRO datasets. The model achieved an accuracy of 98.33 on ModelNet10, 89.31 on ModelNet40 [17], and 99.17 on MIRO datasets.

**Figure 17:**
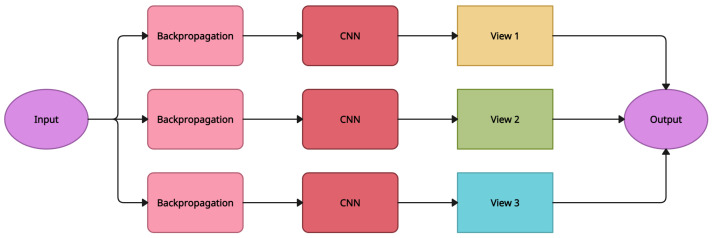
An Overview of the Training Process of RotationNet [22].

Figure 17 shows the RotationNet training procedure. For view rotation, there are three contenders: (1, 2, 3), (2, 3, 1), and (3, 1, 2). By multiplying the histograms for each contender, their score for the ground-truth category is calculated and then the best option in each case is selected. Finally, using the predicted viewpoint variables, the CNN parameters are updated in a conventional back-propagation method.

RotationNet created the dataset Multi-view images of rotated objects (MIRO). The dataset contains 120 object examples divided into 12 categories, with 10 object instances in each. This dataset is solely utilised by RotationNet and has not become a widely used dataset. RotationNet does, however, have the disadvantage of needing each image to be viewed from one of the predetermined views, which is quite limiting when there are fewer predefined viewpoints. RotationNet contains a few predefined viewpoints and demands all views to be input into the network during the training step. Nevertheless, evaluating all perspectives necessitates a significant amount of computing, and not every view is useful for recognition. For feature extraction, RotationNet employs AlexNet [74] as the backbone network, which is smaller than the VGG-M [75] network design. With fewer parameters, it can achieve competitive performance for 3D object retrieval and categorization [78].

### 5.3. PointGCN

**Figure 18:**
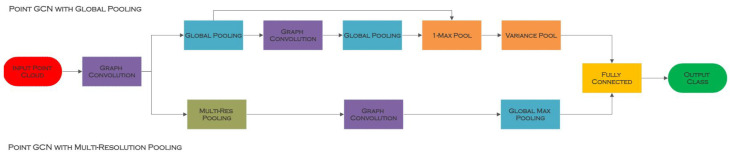
Overview architecture of PointGCN model [8].

A graph-CNN architecture called PointGCN [8] has been designed to categorize 3D point cloud data by examining the local structure that is stored in the created graph. In this scenario, the signals and the graph structure vary depending on the input, and the point cloud data is pooled using two different types of existing graph convolution operations. At various receptive fields, it learns a latent signature that summarizes each point cloud. Figure 18 shows the overall architecture of the PointGCN model. The bottom branch of the image reflects the model architecture utilizing multi-resolution pooling, while the top branch represents the model architecture using only the global pooling layer. It combines the convolutional, pooling, and fully-connected layer types. Two fast localized graph convolutional layers and a layer specifically built to pool point cloud data using global or multi-resolution pooling are included in the model. PointGCN is evaluated on ModelNet10 and ModelNet40 [17] datasets and achieved an accuracy of 91.57 and 89.51, respectively.

PointGCN creates a graph CNN architecture to capture the local structure and categorise point clouds, demonstrating the enormous potential of geometric deep learning for unordered point cloud research [79]. This approach builds a graph structure from the whole 3D point cloud inputs and then filters the spectral graph with filters approximated by Chebyshev polynomials. Nevertheless, because the graph signal is represented by raw 3D coordinates, this technique is still subject to geometric alterations [80]. Unlike point-based approaches, graph-based methods generate a graph-like local area for each point and then feed the graph data into a planned network rather than directly using a discrete point as input. Yet, properly extracting local information from point cloud data’s varied graph topologies remains difficult. In PointGCN, K-NN is utilised to create the local graph by searching for the k-nearest neighbourhood points around the centre point within a given scope. It is incapable of integrating long-distance geometric correlations in a constrained environment, restricting the geometric representation of local points and assisting the point network in capturing more local information [81].

### 5.4. MeshCNN

Irregular triangular meshes are operated on directly by MeshCNN [14], a CNN model created exclusively for meshes, which performs convolution and pooling operations in line with the distinct mesh features. As a mesh’s edges are indented to precisely two faces (triangles), generating a natural fixed-sized convolutional neighborhood of four edges, they are made to resemble pixels in an image. Mesh pooling, which acts on irregular structures and spatially adjusts to the job, is one of the important aspects. Mesh pooling delegated the option of which edges to collapse to the network in a task-specific way, in contrast to standard edge collapse, which eliminates edges that cause a minimal geometric distortion in CNN. MeshCNN can handle different triangulations regardless of the input mesh size and is capable of semantically interpreting both the final output and intermediate computational pooling processes. After pooling, edge pooling is utilised to prevent gaps in the mesh. MeshCNN is evaluated on datasets, like SHREC and COSEG.

Meshes are increasingly being used for learnt geometry and form processing. Despite the fact that mesh-based simulations are the tool of choice in mechanical engineering and related fields, adaptive mesh representations, with a few noteworthy exceptions, have not found considerable usage in machine learning for physics prediction [82]. This approach employs basic linear-mapping transformations and is not resistant to changes in the input [83]. MeshCNN is too expensive to run [84].

### 5.5. InSphereNet

**Figure 19:**
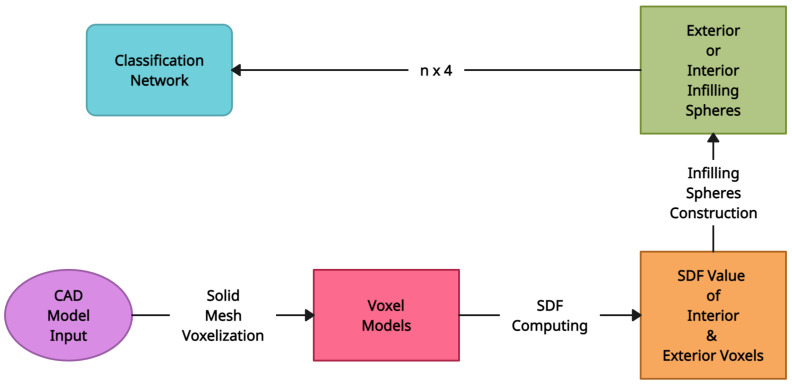
The overall flowchart of InsphereNet model [29].

By extracting Infilling spheres, InSphereNet [29] develops a clear representation and classification approach for 3D object categorization. Spheres with their associated 3D coordinates are built and selected to represent the object as infilling spheres. Space-filling spheres for 3D objects are more instructive and representational than isolated surface points. This is due to the fact that at some points, a surface point is simply identical to a sphere with a radius of zero (surface). A sphere, however, can be found everywhere and in any size. Compared to other techniques of representation, the infilling spheres representation is simpler and more powerful. 3D objects are voxelized with a high resolution of 512 × 512 × 512 voxels and nomalized into a unit size. Four-dimensional vectors are used to build a number of infilling spheres, which are then fed into a simple PointNet network design. InSphereNet is evaluated on ModelNet40 [17] dataset and achieved an accuracy of 90.6. Figure 19 shows the overall flowchart of the InSphereNet model.

Unlike earlier techniques that use point clouds on the surface as DNN inputs, the proposed method can represent 3D shapes from coarse to fine as the number of infilling spheres grows. Experiment findings suggest that InSphereNet outperforms PointNet [57], especially with fewer input features. Even when the number of DNN layers and parameters is reduced significantly, the results are still good. This all suggests that infilling spheres are more representational and relevant than point clouds. One current disadvantage of the suggested strategy is that the infilling spheres remain unstructured.

### 5.6. FPConv

**Figure 20:**
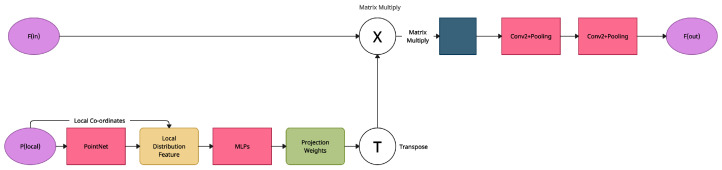
The overall workflow of conducting FPConv on local region centered around point *p* [30].

FPConv [30] is a newly created point cloud convolution procedure that operates directly on the local surface of geometry without using an intermediary grid or graph representation. It operates in a projection-interpolation way, but is more broad and implicit, and the learning process for weight maps may be condensed into a single step. Each point’s convolution weights along the local surface are diffused by FPConv. This significantly enhances the performance of surface-style convolution and makes it more resilient to different input data. By excelling in relatively flat regions, FPConv may be used for 3D object classification and 3D scene semantic segmentation. Figure 20 shows the procedure for performing FPConv on a nearby area centered on point *p*. *N* neighbor points that were randomly selected within a radius range of *p* provide the input coordinates and characteristics, $F_{out}$ at *p* is the output. FPConv is evaluated on ModelNet40 [17] and (S3DIS) [12] datasets and achieved an accuracy of 92.5 and 89.9, respectively.

While FPConv has made significant advances in 3D point cloud processing using deep learning, the task remains challenging due to the sparse, irregular, and unordered nature of point clouds. This point convolution approach employs intricate architecture and data augmentation specific to its operators for assessment, making it difficult to quantify the convolutional operator’s progress. FPConv uses soft weights to flatten local patches onto conventional 2D grids. Nevertheless, it strongly relies on a tangent plane estimate, and the projection procedure will unavoidably compromise 3D geometry information [85].

### 5.7. Global-Local Bidirectional Reasoning (GLR)

GLR [31] is a method for unsupervised point cloud representation learning by using bidirectional reasoning between local representations at various abstraction layers in a network and the global representation of a 3D object. This approach is straightforward, practical, and applicable to a variety of deep learning techniques for interpreting point clouds. With this technique, the underlying semantic information that connects local structures and overall shapes in 3D point clouds will be captured. Both local-to-global and global-to-local reasoning are capabilities of GLR. This model used Relation Shape CNN (RS-CNN), which is insensitive to coordinates and resilient to rigid transformation since it is based on low-level relations rather than coordinates alone. RS-CNN learns the relationships within a small region using geometric priors. For the predefined geometric relation, relation-shape convolution is shape-aware. The relation-shape convolution collects the key contents adaptively based on the weight from the preset function. It then applies a channel-raising mapping after the weighted features for a more powerful shape-aware representation. FPConv is evaluated on ModelNet10, ModelNet40 [17] and (S3DIS) [12] datasets and achieved an accuracy of 95.53, 93.02 and 87.2, respectively [28].

For many reasons, such as different pretraining procedures and differences in feature extractors, a true comparison of this method with other methods is impossible [86]. This approach combined contrastive learning, normal estimation, and self-reconstruction into a single framework, resulting in a multi-task learning system [87]. By bidirectional reasoning between the local structures and the global shape, PointGLR effectively captures the underlying high-level semantic information and achieves improved performance on classification tests. Nevertheless, PointGLR is based on hierarchical local features and is not ideal for networks such as PointNet [57,88]. RS-CNN computes a point feature for early exploration by aggregating information weighted by predetermined geometric relations (low-level relation) between the point and its neighbours. Because of the lack of interaction between features, RS-CNN is inadequate for learning semantic relations (high-level relations). The low-level connection cannot completely capture the relationship between the two places. Relation-shape convolution is useful for learning geometric relations on point clouds, but semantic level relations may be avoided [28].

### 5.8. Rigid Subset Mix (RSMix)

Rigid Subset Mix (RSMix) [32] is a technique for adding data to point clouds that keeps the structure of the original samples while partially mixing two samples. To extract components from each sample, the mask region from the image analysis is redesigned and converted to 3D space. In order to handle unordered structure and non-grid and exploit the structural data of the original point cloud sample, a Rigid Subset (RS) is produced from the redefined mask region. The training sample’s variety and the regularization effects are improved by scaling the RS scale. Since RSMix only uses a portion of the provided data, it may be utilized completely in combination with current data augmentation. By employing RS, the generality of deep neural networks is increased, and emphasis is given to recognizing individual sections of the object. PointGCN is evaluated on ModelNet10 and ModelNet40 [17] datasets and achieved an accuracy of 95.9 and 93.5, respectively.

Point cloud augmentation techniques, which randomly combine points of different forms to produce more diverse shapes, can improve point cloud classification and can be extended to shape segmentation. Random augmentation, on the other hand, does not take form structure into account and can only result in minor improvements [89]. RSMix uses a rigid transformation to combine two point clouds. Yet, as a result of the mixing technique, classifiers become more susceptible to scaling effects [90].

### 5.9. GDANet

**Figure 21:**
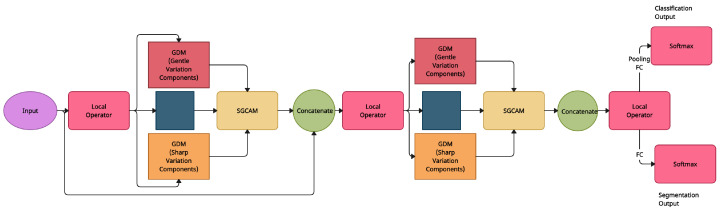
The network architecture of GDANet for Classification and Segmentation [33].

By using Sharp-Gentle Complementary Attention Module (SGCAM) and Geometry-Disentangle Module (GDM), GDANet [33] is able to collect and enhance the comprehensive and complementary geometries of 3D objects to enhance nearby local information. The SGCAM is made to focus on and fuse each original point feature with features from sharp and gentle variation components using geometric correlation. The GDM factors the original point cloud into the contour and flat sections of objects by analyzing graph signals on 3D point clouds at various semantic levels. The GDANet’s network architecture deconstructs the original point cloud and merges features into input point features. Figure 21 shows the network architecture of GDANet. GDANet was evaluated on ModelNet40 [17] and got an accuracy of 93.8 outperforming FPConv [30] which has an accuracy of 92.5. On ScanObjectNN [20] dataset, the model was able to achieve an accuracy of 88.5.

To integrate local features, this technique employs hierarchical multi-scale or weighted feature aggregation algorithms. Despite this, they all use the same MLPs to convert point characteristics, limiting the model’s ability to capture spatial-variant information [85]. This method creates sophisticated grouping strategies like Frequency Grouping to include structural prior into architecture design. Frequency grouping groups point characteristics in the frequency domain using a graph high-pass filter. Still, it is worth noting that advanced grouping takes more time during both training and assessment [90].

### 5.10. Point Transformer

**Figure 22:**
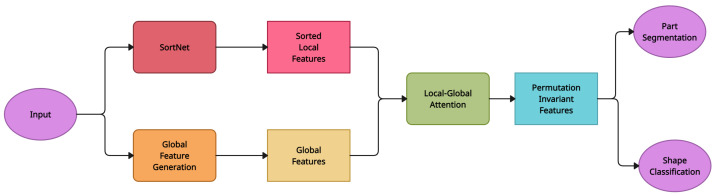
Overview of Point Transformer that outputs permutation invariant and sorted feature set [34].

By learning significant key points or top-k picks, SortNet [34], a permutation invariant network module, learns ordered subsets of the input with latent properties of local geometric and spatial interactions. The set pooling procedure is replaced by these top-k picks. As the global features of the whole point cloud are coupled to the sorted local features via local global attention, which attends both feature representations to capture the underlying form, SortNet is used to create local features of the point cloud. Since the local features are ordered, the output of local-global attention is also ordered and permutation invariant, making it helpful for visual tasks like shape classification and part segmentation as shown in Figure 22. Point Transformer was evaluated on ModelNet40 [17] and got an accuracy of 92.8.

While this approach may readily take use of rich local geometry information and typically produce promising results, it is hampered by two limitations. First of all, the inclusion of delicate extractors significantly increases computing complexity, resulting in prohibitive inference delay. Consequently, with the introduction of local feature extractors, the performance increase on prominent benchmarks has begun to saturate. These restrictions motivate users to devise innovative ways that avoid the need for complex local extractors while producing satisfying results [44].

## 6. Conclusions

Due to their outstanding results in 2D computer vision, deep learning models have quickly emerged as a prominent approach for 3D recognition problems. Many new deep-learning models have been developed and evaluated against various benchmark datasets in the field of object recognition. In order to provide the researchers with a better understanding of these domains, developments of recent 3D data-based object segmentation, detection, and classification systems are discussed in this survey. Different techniques are extensively reviewed, and the efficiency of these methods is calculated based on a selection of commonly used datasets. This survey has also briefly discussed the most popular pipelines, examined their distinctive traits, and assessed how various object recognition strategies differ from one another.

To further explore the potential of these networks, it would be beneficial for future studies to investigate their performance on a wider range of 3D datasets. Additionally, it is important to consider inference time as a key factor in optimizing these networks. This research study [21] has shown that efficiently extracting bounding boxes before segmentation can significantly reduce inference time. These findings can serve as a foundation for future deep learning-based models for 3D object recognition. However, some current models still require tuning and consume more RAM. To address these issues, it will be crucial to develop an efficient model that is fully optimized for speed and memory usage. Therefore, future studies should focus on improving detection and localization accuracy, as well as exploring the fusion of LiDAR voxel features with image features. This includes investigating joint camera-based detection and LiDAR-based detection methods.

## Figures and Tables

**Table 1:** Some LiDAR-based 3D Object Recognition Methods included in this Survey.

Modality	Method Category	Methods
LiDAR-based	Voxel-based	VoxelNet [1], SECOND [2]
LiDAR-based	Point-based	PointRCNN [3], STD [4], PointPillars [5], SA-SSD [6], SLidR [7]
LiDAR-based	Graph-based	PointGCN [8]
LiDAR-Camera Fusion	Multi-view	AVOD [9]

**Table 2:** Benchmaring Datasets included in this survey.

Datasets	Number of Frames	Number of Labels	Object Type	5 Common Classes	URL
KITTI 3D Object Detection [16]	12,000	40,000	Scans of autonomous driving platform	Car, Cylclist, Pedestrian, Tram, Van	https://www.cvlibs.net/datasets/kitti/ (accessed on: 1 February 2023)
SemanticKITTI [11]	43,000	25	Scans from KITTI Vision odometry	Bicycle, Bicyclist, Building, Car, Fence	http://www.semantic-kitti.org/dataset.html (accessed on: 1 February 2023)
ModelNet [17]	151,128	660	3D CAD scans	Bed, Chair, Desk, Sofa, Table	https://modelnet.cs.princeton.edu/ (accessed on: 1 February 2023)
S3DIS [12]	271	12	Scans of restrooms, lobbies, stairways, hallways	Beam, Board, Chair, Door, Sofa	http://buildingparser.stanford.edu/ (accessed on: 1 February 2023)
nuScene [18]	1000	23	Scans of autonomous driving platform	Bicycle, Car, Lane, Stop Line, Walkaway	https://nuscenes.org/ (accessed on: 1 February 2023)
ScanNet [19]	2,492,518	1513	Scans of bedrooms, kitchen, offices	Bed, Chair, Desk, Door, Floor	http://www.scan-net.org/ (accessed on: 1 February 2023)
ScanObjectNN [20]	15,000	2902	Scans of bedrooms, kitchen, offices	Bag, Bed, Bin, Box, Desk	https://hkust-vgd.github.io/scanobjectnn/ (accessed on: 1 February 2023)

**Table 3:** Average Precision (AP) comparison of different 3D object recognition algorithms in the car class of KITTI 3D validation set with IoU threshold 0.7.

Models	Dataset	Average Precision (AP)	IoU Threshold
VoxelNet [1]	KITTI 3D Object Detection [16]	81.97	0.7
AVOD [9]	KITTI 3D Object Detection [16]	84.41	0.7
SECOND [2]	KITTI 3D Object Detection [16]	87.43	0.7
PointRCNN [3]	KITTI 3D Object Detection [16]	88.88	0.7
STD [4]	KITTI 3D Object Detection [16]	89.7	0.7
3DSSD [23]	KITTI 3D Object Detection [16]	89.71	0.7
SA-SSD [6]	KITTI 3D Object Detection [16]	90.15	0.7
PointPillars [5]	KITTI 3D Object Detection [16]	90.19	0.7
FuDNN [24]	KITTI 3D Object Detection [16]	92.48	0.7

**Table 4:** Accuracy comparison of different 3D object recognition algorithms on ModelNet10 dataset.

Models	Dataset	Accuracy
PointGCN [8]	ModelNet10 [17]	91.91
GLR [31]	ModelNet10 [17]	95.53
RSMix [32]	ModelNet10 [17]	95.9
RotationNet [22]	ModelNet10 [17]	98.46

**Table 5:** Accuracy comparison of different 3D object recognition algorithms on ModelNet40 dataset.

Models	Dataset	AP
PointGCN [8]	ModelNet40 [17]	89.51
InSphereNet [29]	ModelNet40 [17]	92.1
FPConv [30]	ModelNet40 [17]	92.5
Point Transformer	ModelNet40 [17]	92.8
GLR [31]	ModelNet40 [17]	93.02
RSMix [32]	ModelNet40 [17]	93.5
GDANet [33]	ModelNet40 [17]	93.8
RPNet [28]	ModelNet40 [17]	94.1
RotationNet [22]	ModelNet40 [17]	97.37

**Table 6:** Mean-per-IoU (mIoU) comparison of different 3D object recognition algorithms on S3DIS dataset.

Models	Dataset	Mean Per-Class IoU (%)
FPConv [30]	S3DIS [12]	66.7
GRA [28]	S3DIS [12]	70.8

**Table 7:** Accuracy comparison of different 3D object recognition algorithms on ScanObjectNN dataset.

Models	Dataset	Accuracy
GLR [31]	ScanObjectNN [20]	87.2
GDANet [33]	ScanObjectNN [20]	88.5

**Table 8:** 3D Segmentation Methods included in this survey.

Models	Technology	Datasets Used	BackBone
3D-CNN [25]	Bird’s Eye View (BEV) projection	SemanticKITTI [11]	2DCNN
RPNet [28]	Group Relation Aggregator (GRA)	ModelNet40 [17], ScanNet [19], S3DIS [12]	PointNet++ [36]
HiLo [21]	Semantic Segmentation	3D-CT	CNN, O-Net
Swin UNETR [37]	Semantic Segmentation	Multi-modal Brain Tumor Segmentation Challenge (BraTS) [38]	Swin Transformer
Meta-RangeSeg [15]	Range Residual Image	SemanticKITTI [11]	U-Net
SLiDR [7]	Image-to-LiDAR Self-supervised Distillation	nuScenes [18], SemanticKITTI [11]	U-Net

**Table 9:** Advantages and Limitations of 3D Segmentation Methods included in this survey.

Models	Technology	Advantages	Limitations
3D-CNN [25]	Bird’s Eye View (BEV) projection	Addressed the issue of Occlusion by using deep learning to fill in the occluded parts	This approach depends heavily on voxel-wise completion labels and perform poorly on little, distant objects and cluttered scenes
RPNet [28]	Group Relation Aggregator (GRA)	Uses relations to learn from local structural information essential for learning point cloud information	Non-convolutional as the input of it’s MLPs contains the absolute location of the points
HiLo [21]	Semantic Segmentation	Can successfully separate firearms within baggage	None of the evaluated super-resolution O-Net topologies can attain the necessary results
Swin UNETR [37]	Semantic Segmentation	Computes self-attention via an efficient shifting window partitioning algorithm and ranks first on the BraTs 2021 validation set [38]	Requires a swin transformer to extract and down-sample feature maps before feeding them into a transformer
Meta-RangeSeg [15]	Range Residual Image	This technique can handle the problem of hazy segmentation borders	Requires boundary loss function to handle the problem of hazy segmentation borders
SLiDR [7]	Image-to-LiDAR Self-supervised Distillation	Pre-training process does not require any annotation of the images nor of the point clouds	Heavily reliant on a huge collection of annotated point clouds

**Table 10:** 3D Detection Methods included in this survey.

Models	Technology	Datasets Used	BackBone
VoxelNet [1]	Voxel Feature Encoding	KITTI 3D Object Detection [16]	PointNet [57], Regional Proposal Network (RPN)
SECOND [2]	Sparse Convolution	KITTI 3D Object Detection [16]	Sparse Convolution, Regional Proposal Network (RPN)
PointPillars [5]	Pointcloud to Pseudo-Image Conversion	KITTI 3D Object Detection [16]	2DCNN
SA-SSD [6]	Feature Map Warping	KITTI 3D Object Detection [16]	Auxiliary Network (CNN)
STD [4]	Proposal Feature Generation	KITTI 3D Object Detection [16]	PointNet++ [36]
PointRCNN [3]	Bottom-Up 3D Proposal Generation	KITTI 3D Object Detection [16]	PointNet++ [36]
3DSSD [23]	Fusion of D-FPS and F-FPS	KITTI 3D Object Detection [16], nuScenes [18]	Multi-Layer Perceptron (MLP)
IMVoteNet [13]	Reformulated Hough Voting	SUN RBG-D	PointNet++ [36]
AVOD [9]	Multimodal Feature Fusion	KITTI 3D Object Detection [16]	Feature Fusion Regional Proposal Network (RPN)
FuDNN [24]	Attention-based Fusion	KITTI 3D Object Detection [16]	2DCNN, Region Proposal Network (RPN)

**Table 11:** Advantages and Limitations of 3D Detection Methods included in this survey.

Models	Technology	Advantages	Limitations
VoxelNet [1]	Voxel Feature Encoding	Demonstrates that switching from a box representation to a center-based representation results in a 3-4 mAP boost	Requires a 3D encoder to quantize the point-cloud into regular bins
SECOND [2]	Sparse Convolution	Streamlines the VoxelNet and accelerates sparse 3D convolutions	Similar to VoxelNet [1], a 3D encoder is used that adds needless costs when selecting thresholds for different classes or datasets
PointPillars [5]	Pointcloud to Pseudo-Image Conversion	Demonstrated a lidar-only solution that outperformed many previous fusion-based algorithms. Quickest recorded method in terms of inference time	More effort is required to integrate multimodal measures in a principled way
SA-SSD [6]	Feature Map Warping	This work enhances feature representation by utilising auxiliary tasks without incurring additional computing burden during inference	Similar to VoxelNet [1] and SECOND [2], a 3D encoder is required to quantize the point-cloud into regular bins
STD [4]	Proposal Feature Generation	Uses a refinement network that is completely independent of the previous pipeline step, which provides more alternatives in terms of training and testing methodologies, resulting in better results	Increases inference time
PointRCNN [3]	Bottom-Up 3D Proposal Generation	Extracts discriminative features directly from raw point clouds for 3D detection	Suffers from the sparse and non-uniform point distribution, as well as the time-consuming process of sampling and searching for nearby points
3DSSD [23]	Fusion of D-FPS and F-FPS	Achieves a good combination of accuracy and efficiency	Suffers from the sparse and non-uniform point distribution, as well as the time-consuming process of sampling and searching for nearby points
IMVoteNet [13]	Reformulated Hough Voting	Primarily based on the set abstraction operation, which permits adjustable receptive fields for learning point cloud features	Depends on Non-Maximal Suppression (NMS) as a post-processing step to eliminate the loss
AVOD [9]	Multimodal Feature Fusion	Converts irregular point clouds to 2D bird-view maps, which may then be effectively processed by 3D or 2D CNN to train point features for 3D detection	Hard-coded feature extraction method may not extend to new setups without substantial engineering work
FuDNN [24]	Attention-based Fusion	Creates 3D region proposals based on a bird’s-eye view and conducts 3D bounding box regression	Texture information in the picture data may not be properly exploited.

**Table 12:** 3D Classification Methods included in this survey.

Models	Technology	Datasets Used	BackBone
OctNet [10]	Hybrid Grid-OctTree Data Structure	ModelNet10 [17]	U-shaped Network
RotationNet [22]	Pose Estimation from Multi-View Images of an Object	ModelNet10 [17], ModelNet40 [17], MIRO	CNN
PointGCN [8]	Graph Convolutions and Graph Downsampling Operations	ModelNet10 [17], ModelNet40 [17]	GCN
MeshCNN [14]	Convolution, Poling and Unpooling of Mesh	SHREC, COSEG	CNN
InSphereNet [29]	Signed Distance Field (SDF) Computation	ModelNet40 [17]	MLP
FPConv [30]	Flattening Projection Convolution	ModelNet40 [17], S3DIS [12]	2DCNN
GLR [31]	Unsupervised Feature Learning	ModelNet10 [17], ModelNet40 [17], ScanObjectNN [20]	PointNet++ [36], Relation-Shape CNN (RSCNN)
RSMix [32]	Shape-preserving Data Augmentation	ModelNet10 [17], ModelNet40 [17]	Pointnet++ [36], DGCNN
GDANet [33]	Geometry Disentanglement	ModelNet40 [17], ScanObjectNN [20]	GDM, SGCAM
Point Transformer [34]	Local-Global Attention Mechanism	ModelNet40 [17]	SortNet

**Table 13:** Advantages and Limitations of 3D Classification Methods included in this survey.

Models	Technology	Advantages	Limitations
OctNet [10]	Hybrid Grid-OctTree Data Structure	Employs octrees that allows for wider grids and improved speed	Octrees are imbalanced and have hierarchical divisions. This network lacks flexibility because its kernels are limited to 27 or 125 voxels
RotationNet [22]	Pose Estimation from Multi-View Images of an Object	Employs AlexNet [74] as the backbone network, which is smaller than the VGG-M [75] network design and can achieve competitive performance for 3D object retrieval and categorization	Needs each image to be viewed from one of the predetermined views, which is quite limiting when there are fewer predefined viewpoints. Evaluating all perspectives necessitates a significant amount of computing, and not every view is useful for recognition.
PointGCN [8]	Graph Convolutions and Graph Downsampling	Creates a graph CNN architecture to capture local structure and categorise point clouds, demonstrating the enormous potential of geometric deep learning for unordered point cloud research	K-NN is utilised which is incapable of integrating long-distance geometric correlations in a constrained environment, restricting the geometric representation of local points and assisting the point network in capturing more local information
MeshCNN [14]	Convolution, Pooling and Unpooling of Mesh	Works on meshes that are increasingly being used for learnt geometry and form processing	Mesh-based simulations have not found considerable usage in machine learning for physics prediction. Too expensive to run.
InSphereNet [29]	Signed Distance Field (SDF) Computation	Outperforms PointNet [57] especially when the number of DNN layers and parameters are reduced significantly, the results are still good	Infilling spheres remain unstructured
FPConv [30]	Flattening Projection Convolution	Uses soft weights to flatten local patches onto conventional 2D grids	Strongly relies on tangent plane estimate, and the projection procedure will unavoidably compromise 3D geometry information
GLR [31]	Unsupervised Feature Learning	Effectively captures the underlying high-level semantic information and achieves improved performance on classification tests	Based on hierarchical local features and is not ideal for networks such as PointNet
RSMix [32]	Shape-preserving Data Augmentation	Point cloud augmentation techniques can improve point cloud classification and can be extended to shape segmentation	Uses rigid transformation to combine two point clouds making classifiers become more susceptible to scaling effects
GDANet [33]	Geometry Disentanglement	Creates sophisticated grouping strategies like Frequency Grouping to include structural prior into architecture design	Frequency grouping takes more time during both training and assessment
Point Transformer [34]	Local-Global Attention Mechanism	SortNet is used to generate point cloud local features making the output of local-global attention ordered and permutation invariant. This makes it useful for visual tasks such as form classification and part-segmentation.	Inclusion of delicate extractors significantly increases computing complexity, resulting in prohibitive inference delay. With the introduction of local feature extractors, the performance increase on prominent benchmarks has begun to saturate.

## Data Availability

Not applicable.

## Abbreviations

The following abbreviations are used in this manuscript:

3D	three-dimensional
2D	two-dimensional
LiDAR	Light Detection And Ranging
RGB-D	Red, Green, Blue plus Depth
CAD	Computer-Aided Design
MLP	Multiple Layer Perceptron
BEV	Bird’s Eye View
CNN	Convolutional Neural Network
GRA	Group Relation Aggregator
GPU	Graphics Processing Unit
RAM	Random Access Memory
MRI	Magnetic Resonance Imaging
SECOND	Sparsely Embedded CONvolutional Detection
IoU	Intersection over Union
RPN	Region Proposal Network
GLR	Global-Local Bidirectional Reasoning
